# Integrated Positron Emission Tomography/Magnetic Resonance Imaging for Resting-State Functional and Metabolic Imaging in Human Brain: What Is Correlated and What Is Impacted

**DOI:** 10.3389/fnins.2022.824152

**Published:** 2022-03-02

**Authors:** Yi Shan, Zhe Wang, Shuangshuang Song, Qiaoyi Xue, Qi Ge, Hongwei Yang, Bixiao Cui, Miao Zhang, Yun Zhou, Jie Lu

**Affiliations:** ^1^Department of Radiology and Nuclear Medicine, Xuanwu Hospital, Capital Medical University, Beijing, China; ^2^Beijing Key Laboratory of Magnetic Resonance Imaging and Brain Informatics, Beijing, China; ^3^Central Research Institute, United Imaging Healthcare Group, Shanghai, China

**Keywords:** PET, fMRI, brain metabolism, integrated PET/MRI, quantification analysis

## Abstract

Integrated positron emission tomography (PET)/magnetic resonance imaging (MRI) could simultaneously obtain both functional MRI (fMRI) and ^18^F-fluorodeoxyglucose (FDG) PET and thus provide multiparametric information for the analysis of brain metabolism. In this study, we aimed to, for the first time, investigate the interplay of simultaneous fMRI and FDG PET scan using a randomized self-control protocol. In total, 24 healthy volunteers underwent PET/MRI scan for 30–40 min after the injection of FDG. A 22-min brain scan was separated into MRI-off mode (without fMRI pulsing) and MRI-on mode (with fMRI pulsing), with each one lasting for 11 min. We calculated the voxel-wise fMRI metrics (regional homogeneity, amplitude of low-frequency fluctuations, fractional amplitude of low-frequency fluctuations, and degree centrality), resting networks, relative standardized uptake value ratios (SUVr), SUVr slope, and regional cerebral metabolic rate of glucose (rCMRGlu) maps. Paired two-sample *t*-tests were applied to assess the statistical differences between SUVr, SUVr slope, correlation coefficients of fMRI metrics, and rCMRGlu between MRI-off and MRI-on modes, respectively. The voxel-wise whole-brain SUVr revealed no statistical difference (*P* > 0.05), while the SUVr slope was significantly elevated in sensorimotor, dorsal attention, ventral attention, control, default, and auditory networks (*P* < 0.05) during fMRI scan. The task-based group independent-component analysis revealed that the most active network components derived from the combined MRI-off and MRI-on static PET images were frontal pole, superior frontal gyrus, middle temporal gyrus, and occipital pole. High correlation coefficients were found among fMRI metrics with rCMRGlu in both MRI-off and MRI-on mode (*P* < 0.05). Our results systematically evaluated the impact of simultaneous fMRI scan on the quantification of human brain metabolism from an integrated PET/MRI system.

## Introduction

Positron emission tomography (PET) with ^18^F-fluorodeoxyglucose (FDG) has been regarded as the gold-standard technique to quantify brain energy metabolism. Despite standardized uptake values (SUV) reflecting regional FDG uptake, intrinsic networks derived from FDG-PET using independent-component analysis (ICA) could reflect long-distance metabolic brain connections. Based on this data-driven technique, the characteristics of metabolic resting-state networks (RSN) could be utilized to observe a cognitive decline in Alzheimer’s disease and amyotrophic lateral sclerosis (Pagani et al., 2016, 2017). Another promising aspect of FDG-PET is to couple glucose utilization with cerebral blood flow and oxygen consumption. Blood-oxygenation-level-dependent (BOLD) functional magnetic resonance imaging (fMRI) has been commonly used to non-invasively obtain oxygen metabolism and hemodynamic response related to brain activity (Bernier et al., 2017). The neurometabolic coupling between fMRI and FDG PET metrics has provided informative biomarkers for characterizing focal differences of energy demand in a healthy population and pathology processes such as alcohol exposure and disorders of consciousness (Soddu et al., 2016; Tomasi et al., 2017; Shokri-Kojori et al., 2019).

The integrated PET/MRI system could simultaneously obtain both fMRI and FDG PET images (Disselhorst et al., 2014; Chen et al., 2018). With the advantage of precisely matched structural localization and consistent physiological states, the combined system could provide a great opportunity for multiparametric analysis of interactions between brain metabolism and function (Heiss, 2009; Verger and Guedj, 2018). With the addition of FDG PET, fMRI showed potential for baseline shifts in quantifiable metabolism and neuronal signaling (Riedl et al., 2014, 2016). Also, with the fusion of fMRI, FDG PET could track dynamic task-related hemodynamic and metabolic interactions with higher temporal resolution (Jamadar et al., 2019). Recently, the integration of the two modalities has proved to be cost-effective for clinical research, especially in neurodegenerative disorders. Focal alterations between FDG uptake and fMRI metrics obtained by integrated PET/MRI showed potential to reveal signaling hierarchies in hippocampal–cortical circuits and default mode networks in patients with Alzheimer’s disease (Tahmasian et al., 2015, 2016; Marchitelli et al., 2018; Scherr et al., 2019; Yan et al., 2020). Altered bioenergetic coupling across the gray matter and its relationship with seizure outcomes were also reported in patients with medial temporal lobe epilepsy (Wang et al., 2020). Thus, simultaneous PET/fMRI has become a widely adopted approach for non-invasive brain metabolic research.

In order to shorten the time of scan, some clinical research using the integrated PET/MRI system prefer to run MRI sequences during the FDG uptake phase (the first 40 min after the radiotracer administration). However, the acoustic, thermal, and electromagnetic effect imposed by MRI on the human brain may result in physiological inference on quantitative FDG uptake curve. Although it has been assessed on phantoms that the stability of PET quantitation was not affected during simultaneous MRI scan, even when an aggressive sequence such as fast-spin echo MRI sequence was performed (Grant et al., 2016; Deller et al., 2018; Chen et al., 2021), the impact of MRI sequence pulsing was found in human studies to affect brain activation, including auditory, visual, and motor functional cortex (Di Salle et al., 2001; Tomasi et al., 2005; Zhang et al., 2005; Fuchino et al., 2006; Gaab et al., 2008; Andoh et al., 2017). A recent study has shown that the acoustic noise produced by fast switching gradients could cause a reproducible increase ranging from 3 to 9% in FDG uptake, which is limited to the primary auditory cortex (Chonde et al., 2013). Therefore, the timing of the fMRI scan is preferred to be run during the plateau phase (30–40 min after the radiotracer administration) of the FDG uptake curve in clinical routine. During this period, the most irreversible FDG distribution is considered to be finished with only a minimal amount of free FDG in the blood pool. However, few studies have proven that the impact induced by MRI scan during this “static” period could be limited to an acceptable range in human brain. Also, whether the possible effect could influence a specific metabolic parameter or neurometabolic coupling limited to the focal brain cortex, network level or whole-brain level, is largely unknown.

Therefore, the principal purpose of this work is to systematically evaluate the impact of simultaneous fMRI scan on the quantification of brain metabolism using an integrated PET/MRI system. We designed a randomized self-control study on healthy volunteers using clinically relevant imaging protocols. We will focus on the additional effect induced by fMRI sequence pulsing on voxel-wise energy consumption, spatial distribution of metabolic networks as well as the correlation between regional glucose metabolism and functional metrics within and across different resting-state functional networks.

## Materials and Methods

### Subjects

All subjects were provided with written informed consent to undergo procedures approved by the Medical Research Ethics Committee of Xuanwu Hospital, Capital Medical University (Beijing, China). A total of 24 healthy subjects (male, 14; female, 10) with ages of 31–66 years were enrolled in this study. The subjects were all right-handed and free of personal or family history of psychiatric or neurological disease, diabetes, renal failure, claustrophobia, and other MRI-related exclusion criteria. Prior to the scan, the participants were directed to fast for at least 6 h before the ^18^F-FDG injection, with a dosage of 3.7 MBq/kg per subject. Immediately before the scanning began, all subjects underwent a blood glucose level measurement, and all measured values were below 8 mmol/L.

### Data Acquisition Protocol

**FIGURE 1:**
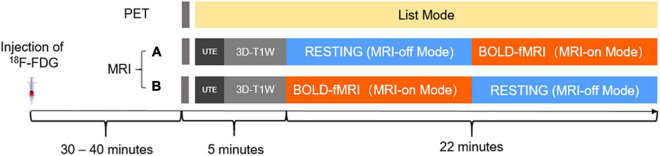
Schematic diagram of the simultaneous ^18^F-FDG PET/MRI acquisition protocol. ^18^F-FDG, ^18^F-2-fluro-D-deoxy-glucose; UTE, ultra-short echo; 3D-T1W, three-dimensional T1-weighted sequence.

Simultaneous ^18^F-FDG PET/MRI scanning was performed on an integrated PET/MRI system (uPMR 790, United Imaging Healthcare, Shanghai, China) equipped with a 24-channel phase-array head coil (Chen et al., 2021). Phantom experiments were initially performed to evaluate the hardware-related effects, the SUV and MRI signal stability of this integrated system (see Supplementary Material). The MRI and PET list mode data were simultaneously acquired 30–40 min after the injection of ^18^F-FDG as illustrated in Figure 1. The subjects were scanned without their ears occluded by earplugs.

The MRI scan started with 5 min of scanning for attenuation correction and 3D anatomical localization, followed by a 22-min experimental scan. Specifically, the first 5 min of MRI scanning consisted of a localizer and an ultra-short echo time MRI sequence for PET attenuation correction and a three-dimensional T1 weighted fast-spoiled gradient echo sequence (voxel sizes = 1.0 mm × 1.0 mm × 1.0 mm) for brain segmentation. The following 22-min experimental scan was separated into MRI-off and MRI-on modes, with each one lasting for 11 min. In the MRI-off mode, no MRI sequence was performed. In the MRI-on mode, a gradient-echo echo-planar pulse sequence (repetition time = 3,000 ms, echo time = 30 ms, flip angle = 90°, number of slices = 43, voxel sizes = 3.0 mm × 3.0 mm × 3.0 mm, matrix size = 64 × 64) was performed for resting-state BOLD-fMRI measurements lasting for 10 min. In order to fairly evaluate the impact of BOLD-fMRI on FDG-PET, all subjects were divided randomly into one of two groups who received either protocol A or B. For the protocol A group, the MRI-off mode was performed ahead of the resting-fMRI, while for the protocol B group, the scan order was reversed (see Figure 1).

The PET list mode data had a 511-KeV energy drift correction applied to correct the temperature-induced counting loss during MRI scanning (Grant et al., 2016). Dynamic data was reconstructed to 10 PET image frames [2 min per frame, matrix size = 192 × 192, field of view (FOV) = 35 cm, voxel size = 1.82 mm × 1.82 mm × 2.78 mm] using the ordered subset-expectation maximization (OSEM) algorithm [three iterations and 20 subsets with time-of-flight (TOF) and point-spread function (PSF)]. Corrections were applied for random coincidences, dead time, scatter, and attenuation. Static data was reconstructed using all list mode events obtained during the 22-min experimental scan (matrix size = 256 × 256, FOV = 25 cm, slice thickness = 1.4 mm, OSEM = 3 iterations and 20 subsets with TOF and PSF).

### Data Processing

#### Data Processing for Functional Maps

**FIGURE 2:**
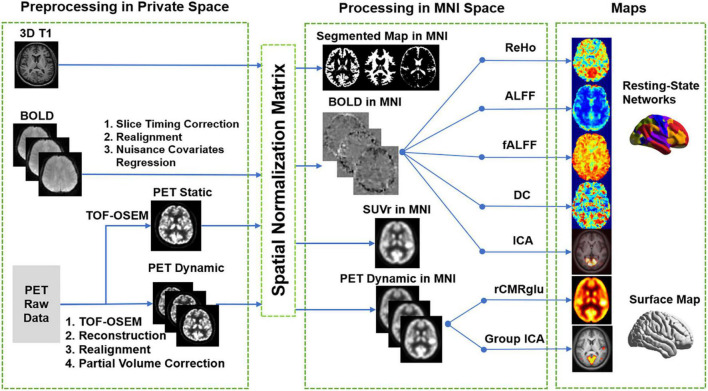
Schematic diagram of data processing protocol. The calculation of SUVr, fMRI metrics, metabolic networks, and rCMRGlu maps were illustrated.

Data on post-processing for PET and MRI images is summarized in Figure 2. The PET and MRI images were registered and jointly processed using SPM12 (Statistical Parametric Mapping)^1^.

#### Generation of Resting-State Functional Metrics From Blood-Oxygenation-Level-Dependent-Functional MRI

The pre-processing of resting-fMRI was performed using the Data Processing and Analysis for Brain Image (DPABI) tool^2^ (Yan et al., 2016). After slice timing correction, alignment correction was applied to reduce the impact of head motion. The fMRI images were spatially transformed to the characteristic T1-weighted structural template. The transformed fMRI images were resampled to 3 mm × 3 mm × 3 mm, and the nuisance covariates (24 head motion parameters, cerebrospinal fluid signal, white matter signal, and linear trend) were regressed out. Voxel-wise fMRI metric maps were generated, including regional homogeneity (ReHo), amplitude of low-frequency fluctuations (ALFF), fractional amplitude of low-frequency fluctuations (fALFF), and degree centrality (DC), using the DPARSF package inside DPABI. Eight resting-state networks (including visual, default, dorsal attention, auditory, ventral attention, control, sensorimotor, and limbic networks) were generated from the same corrected fMRI images using a group ICA analysis package^3^ and were used to mask out the regions of interest (ROIs). The mean value of each metric was calculated in each of the eight ROIs.

### Calculation of Parametric Images From ^18^F-Fluorodeoxyglucose-Positron Emission Tomography

After normalization of focal FDG uptake for body weight and injected dose, the relative standardized uptake value ratios (SUVr) with reference to the cerebellum and white matter was calculated, respectively. To quantify the change of SUVr during the time course of MRI-on and MRI-off mode acquisitions, SUVr slope was derived by linear fitting of the relevant SUVr values from each 2-min frame images with time. An analysis of regional cerebral metabolic rate of glucose (rCMRGlu) at each voxel was performed (Wang et al., 1993). To allow a consistent dynamic analysis, all PET dynamic images were aligned to the first time-point image and co-registered to the corresponding T1-weighted image. The Spearman correlation coefficient (*R*) between rCMRGlu and fMRI metrics was calculated using the python package SciPy^4^. Across-subject correlation was calculated of each pair of rCMRGlu and ALFF, fALFF, DC, and ReHo at the network level.

### Independent-Component Analysis for ^18^F-Fluorodeoxyglucose-Positron Emission Tomography

Spatial ICA of the pre-processed PET images was performed separately within the MRI-off and MRI-on subgroups using the GIFT toolbox^5^. The optimal number of components of the principal component analysis was set to five, which was estimated using the GIFT dimensionality estimation tool. Group ICA was used to derive task-based regional activation, treating MRI-on mode as a paradigm. First, the mixing matrix was estimated, which has a unique partition for each object. The component graph of each task was calculated by projecting a single task data onto the inverse of the mixing matrix partition. Task-specific time courses and images were used to make group and inter-group inferences (Calhoun et al., 2009). Each spatial map was converted to *Z*-values, and activation maps were visualized using binary masks generated with a threshold of 1.5.

### Statistical Analysis

Statistical analysis was performed using DPABI and Statistical Product and Service Solutions (IBM SPSS, version 20). Paired two-sample *t*-tests were applied to assess the significance of SUV, SUVr, and SUVr slope between MRI-off and MRI-on modes, respectively. The voxel-wise paired *t*-test between whole-brain SUVr in MRI-off mode and MRI-on mode was performed using DPABI. Comparisons within correlation coefficients of functional metrics (ReHo, ALFF, fALFF, and DC) and rCMRGlu among the eight networks between MRI-on mode and MRI-off mode were calculated with one-way ANOVA analysis and paired two-sample *t*-test, respectively.

## Results

### Magnetic Resonance Imaging Impact on ^18^F-Fluorodeoxyglucose Uptake

**FIGURE 3:**
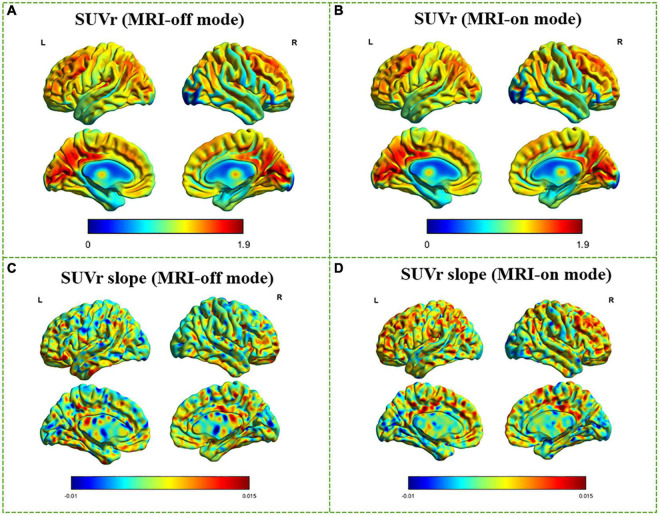
Voxel-wise comparison between MRI-off and MRI-on modes. SUVr and SUVr slope in MRI-off mode **(A,C)** and MRI-on mode **(B,D)** overlaid on three orthogonal views of the brain for a randomly selected subject. Average SUVr and SUVr slope across subjects superimposed on dorsal (top) and medial (bottom) surface views of the cerebrum.

**FIGURE 4:**
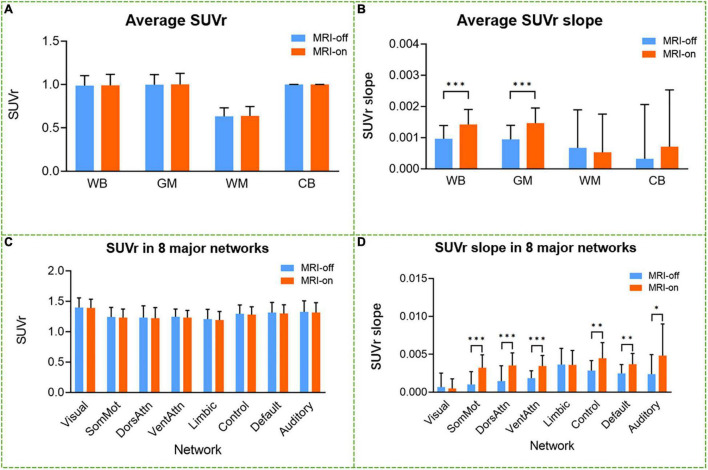
Comparison between average SUV and SUVr slope in MRI-on mode and MRI-off mode. The mean of SUVr **(A,C)** and SUVr slope **(B,D)** was compared across the whole brain, gray matter, white matter, cerebellum, and eight major networks for all the subjects scanned in MRI-off and MRI-on modes. Error bars are standard error. *P-value < 0.05; **P-value < 0.01; ***P-value < 0.001.

The Phantom results have verified the system stability for simultaneous PET/fMRI acquisition (Supplementary Figure 1 and Supplementary Table 1). Static PET images from MRI-off and MRI-on modes produced similar SUVr (SUV_*WB*_/SUV_*CB*_) distribution maps (Figures 3A,B). From visual inspection, concordant distributions were found without focal areas of abnormal hyper- or hypo-metabolism. Voxel-wise paired *t*-tests between whole-brain SUVr in MRI-off mode and MRI-on mode revealed no statistical difference (*P* > 0.05), while the SUVr slope significantly increased when comparing the MRI-on mode with the MRI-off mode (*P* < 0.05, Figures 3C,D). The mean SUVr and SUVr slope values for the whole brain (WB) and different anatomical structures, including grey matter (GM), white matter (WM), cerebellum, and eight functional networks, were calculated (Figure 4). The mean SUVr slope significantly increased in WB and GM, especially in sensorimotor, dorsal attention, ventral attention, control, default, and auditory networks, when comparing MRI-on mode with MRI-off mode, while the mean SUVr showed no statistical difference (*P* > 0.05). The same measurement using white matter as a reference tissue was performed, and a similar result was confirmed (Supplementary Figure 2).

### Magnetic Resonance Imaging Impact on Metabolic Network

**FIGURE 5:**
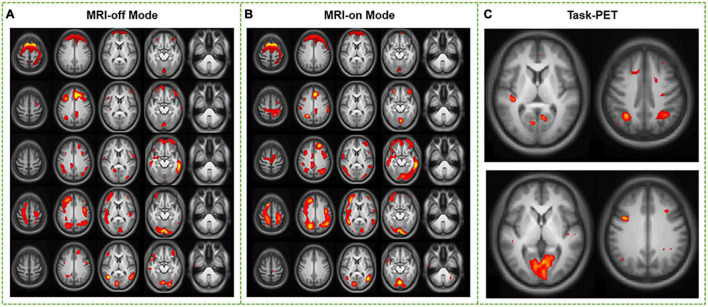
Comparison between static spatial independent-component analysis (ICA) driven from PET data in MRI-off and MRI-on modes. Major networks from resting PET SUVr images in MRI-off and MRI-on modes were persevered **(A,B)**. Regional activation was shown through group ICA of combined MRI-off and MRI-on modes **(C)**.

The most active network components derived from PET static spatial ICA were located in the auditory, default, visual, and language networks (Figures 5A,B). From visual inspection, there was little difference observed between the component maps calculated from the PET image generated during MRI-off and MRI-on modes. The group ICA which treated the fMRI scan as a stimulating paradigm revealed that the most active network components were the frontal pole, superior frontal gyrus, middle temporal gyrus, and occipital pole (Figure 5C).

### Impact of Simultaneous Scan on Neurometabolic Coupling

**FIGURE 6:**
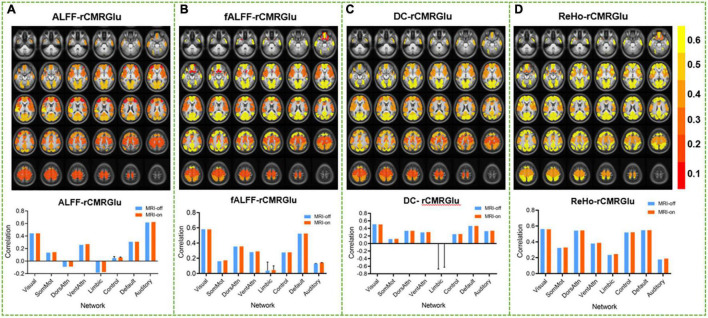
Spatial correlation between regional cerebral metabolic rate of glucose and functional MRI metrics, amplitude of low-frequency fluctuations **(A)**, fractional amplitude of low-frequency fluctuations **(B)**, degree centrality **(C)**, and regional homogeneity **(D)**, in MRI-on mode. The comparison between the corresponding correlation statistics (averaged across all subjects) across the eight networks in the MRI-off and MRI-on modes is shown, respectively.

The spatial correlation maps between rCMRGlu and four fMRI metrics, as well as the results of the correlation analysis over each of the eight resting-state networks, are shown in Figure 6. Overall, high correlation coefficients were found among the four fMRI metrics with rCMRGlu in both MRI-off and MRI-on modes (mean *R* for ALFF, fALFF, DC, and ReHo was 0.195 ± 0.260, 0.296 ± 0.180, 0.287 ± 0.164, and 0.413 ± 0.145, respectively). ReHo provided significantly higher correlation coefficients with rCMRGlu compared to the other metrics (ReHo and ALFF, *P* = 0.013; ReHo and fALFF, *P* < 0.001; ReHo and DC, *P* = 0.002). No difference was found between the other metrics (ALFF and fALFF, *P* = 0.144; fALFF and DC, *P* = 0.686; ALFF and DC, *P* = 0.080). The highest correlation coefficients between rCMRGlu and all fMRI metrics were found in the visual network (mean *R*, 0.523 ± 0.057) and the default network (mean *R*, 0.461 ± 0.099).

## Discussion

To the best of our knowledge, this work is the first study to systematically assess the impact of simultaneous fMRI scan on FDG-PET in human brain with an integrated PET/MRI system. Our protocol is a self-control study design following the recommended clinical routine, where MRI sequences were performed 30–40 min after the injection of ^18^F-FDG. It is commonly considered that the FDG-PET data acquired during this plateau phase of the uptake curve mainly represents the neuronal activity that occurred during the preceding early uptake phase. Thus, SUVr for the plateau phase is supposed to be steady even when fMRI scan is synchronously performed. Our results found no difference in either mean SUVr or voxel-wise SUVr compared between MRI-on and MRI-off periods, which supported this inference. However, an obvious increase of SUVr slope was detected in the MRI-on period across the whole brain, especially in gray matter, located in sensorimotor, attention, control, default, and auditory networks. This network-wise metabolic change reflects a short-term FDG uptake elevation due to the fMRI scan. We assume that this phenomenon was mainly in favor of a neuronal origin whose activation mostly locate in gray matter regions and may also be due to other unspecific physiological inferences, such as the temperature-dependent acceleration of metabolic rates. However, with only a minor amount of free FDG available in the blood pool, fMRI scan in the steady state could only produce a limited, short-term effect on the trend of FDG elevation, which could be presented by the elevation of SUVr slope but does not affect the calculation of an accumulative SUVr value to statistical significance.

In the network-wise comparison, when treating fMRI scan as a stimulation task, the affected components were located in the default, auditory, visual, and language networks, which were commonly regarded as “higher-order” cognitive networks. A previous study reported that 13 meaningful RSNs could be detected from FDG-PET data acquired 10–30 min post-injection. Among them, seven networks could be detected by both modalities, including default mode, left central executive, primary and secondary visual, sensorimotor, cerebellar, and auditory networks (Savio et al., 2017). In our study, the “activated” networks induced by fMRI scan fundamentally located in these “dually” detected RSNs, which could be explained by changes of either cerebral blood flow or activity-dependent glucose consumption. As our data was acquired 30 min post-injection, the contribution of a blood flow signal change caused by the instant injection of FDG could be negligible. In this way, these “activations” observed in our ICA results should be regarded as comparable elevated glucose consumption rather than increased cerebral blood flow or oxygen consumption.

In previous studies, the possible mechanism underlying neurometabolic coupling was explained by temporally synchronized cerebral blood flow and energy utilization based on the theory that resting-state glucose and oxygen metabolism were closely linked (Wang et al., 2021). Strong coupling was found in default and visual networks, while a weak correlation was found in limbic and somatomotor network (Aiello et al., 2015). The highest correlation between rCMRGlu and fMRI metrics was achieved in ReHo, which were both detected by using integrated PET/MRI or separated PET and MRI devices (Aiello et al., 2015; Bernier et al., 2017; Jiao et al., 2019). The other metrics, such as ALFF or DC, showed a lower association with CMRGlu, maybe strongly affected by the venous vasculature or other non-neuronal factors on signal amplitude (Bernier et al., 2017). These differences could also be explained by the different physiological phenomena probed by each metric. ALFF contrast is only due to a single voxel signal. ReHo could be considered as a measurement of short-range functional connectivity (FC) affected by the neighboring 27 voxels, while DC measures distant voxels weighted by long-range FC in the whole brain.

Our study reports a synthesis effect of MRI scan on quantitative PET in an integrated system. Among all possible factors, acoustic MRI noise resulting from echo-planar imaging should be regarded as the main concern. This sequence is normally accompanied by a gradient-shifting noise with a sound level greater than 100 dB (Zhang et al., 2005). Studies have focused on measuring how background acoustic noise influenced the hemodynamic responses in the auditory cortex and made efforts to spoil the interference (Di Salle et al., 2001). Reduced activation in the visual cortex was also reported, which may relate to attention modulation due to auditory–visual cross-modal neural interaction (Zhang et al., 2005). Increased activation of working memory network (Tomasi et al., 2005) and suppressed activation in the default-mode network and sensorimotor cortex (Fuchino et al., 2006; Gaab et al., 2008) were, respectively, discussed under the presence of BOLD-related noise. “Quieter” fMRI acquisition methods, such as sparse temporal sampling or interleaved silent steady state, could be applied to a less noisy background environment for BOLD-fMRI scan (Andoh et al., 2017). In addition, MRI-induced RF power deposition and the resulting effects on temperature-dependent metabolic rates could also influence FDG uptake, with maximum relative increases of 26% for uptake models based on metabolism (Carluccio et al., 2017). We speculate that these abovementioned factors synergistically influenced brain metabolism during the static phase of FDG uptake in our study.

This work was subject to several limitations. First, we adopted a blood-free approach to estimate the relative quantification of CMRGlu, which is more tolerable for a universal clinical routine. However, for a more precise design, absolute quantification of CMRGlu could be calculated by infusion of ^18^F-FDG and venous blood sampling (Hahn et al., 2016). Second, methodologically, ICA and seed-based functional connectivity (sbFC) are two main approaches for the statistical mapping of RSNs derived from FDG-PET. It has been discussed that the choice of ICA or sbFC could influence the detectability of RSNs, especially when the sample size is limited (Trotta et al., 2018). Future studies could retest and verify our results by different data analysis methods based on a larger dataset.

## Data Availability Statement

The raw data supporting the conclusions of this article will be made available by the authors, without undue reservation.

## Ethics Statement

The studies involving human participants were reviewed and approved by the Medical Research Ethics Committee of Xuanwu Hospital, Capital Medical University (Beijing, China). The patients/participants provided their written informed consent to participate in this study.

## Author Contributions

YS, ZW, QX, and YZ contributed to the conception and design of the study. YS, ZW, SS, QX, and QG performed the formal analysis. YS and ZW wrote the first draft of the manuscript. BC and HY contributed to project administration and visualization. JL, YZ, and MZ contributed to supervision and validation. JL contributed to funding acquisition. All authors contributed to manuscript revision and read and approved the submitted version.

## Conflict of Interest

The authors declare that the research was conducted in the absence of any commercial or financial relationships that could be construed as a potential conflict of interest.

## Publisher’s Note

All claims expressed in this article are solely those of the authors and do not necessarily represent those of their affiliated organizations, or those of the publisher, the editors and the reviewers. Any product that may be evaluated in this article, or claim that may be made by its manufacturer, is not guaranteed or endorsed by the publisher.

## Footnotes

1
https://www.fil.ion.ucl.ac.uk/spm/software/spm12/

2
http://www.rfmri.org/dpabi

3
https://www.nitrc.org/projects/gift

4
https://pypi.org/project/scipy/

5
http://mialab.mrn.org/software/
