# Supplementary material for: Integrated Positron Emission Tomography/Magnetic Resonance Imaging for Resting-State Functional and Metabolic Imaging in Human Brain: What Is Correlated and What Is Impacted
Source: Front Neurosci. 2022 Mar 2;16:824152. doi: 10.3389/fnins.2022.824152 (PMC8926297; doi:10.3389/fnins.2022.824152)
Supplement: Supplementary file 1 [file Data_Sheet_1.docx]

Supplementary Material

# Phantom Experiments

To quantitatively evaluate of the SUV and MRI signal stability during simultaneous PET/MR acquisition, a NEMA standard cylinder phantom (diameter 20cm, length 30cm) was filled with 2mCi FDG and scanned at all MRI pulse sequences prescribed for in vivo scans including T2-weightedfast spin echo (FSE), T2-weightedfluid attenuated inversion recovery (FLAIR), echo planar imaging (EPI) based blood oxygen level-dependent (BOLD), diffusion weighted imaging (DWI) and diffusion tensor imaging (DTI). The PET data was acquired in list mode and reconstructed into dynamic frames with a temporal resolution of 2 minutes using the identical protocol as used in in vivo experiments. The mean of SUV was calculated from automatically positioned ROI on the center slice of uniform phantom. MRI image acquisition was repeated with PET turned on and off. Average signal noise ratio (SNR) difference was calculated as （SNR_PETon_– SNR_PEToff_)/SNR_PEToff_×100%.

# System Stability of Integrated PET/MR

PET image of NEMA phantom Image demonstrates the stability of PET quantitation under the noise of fMRI and clinical sequences(Fig. S1). The mean, standard deviation and variance of SUV within all dynamic frames is 1.0023, 0.0087 and 0.0001respectively. In addition, PET operation presented limited impact on SNR ofMRI during the acquisition using clinical and functional MRI sequences used in this study (Table S1). The measured difference between PET on and off is less than 1.06%.

**
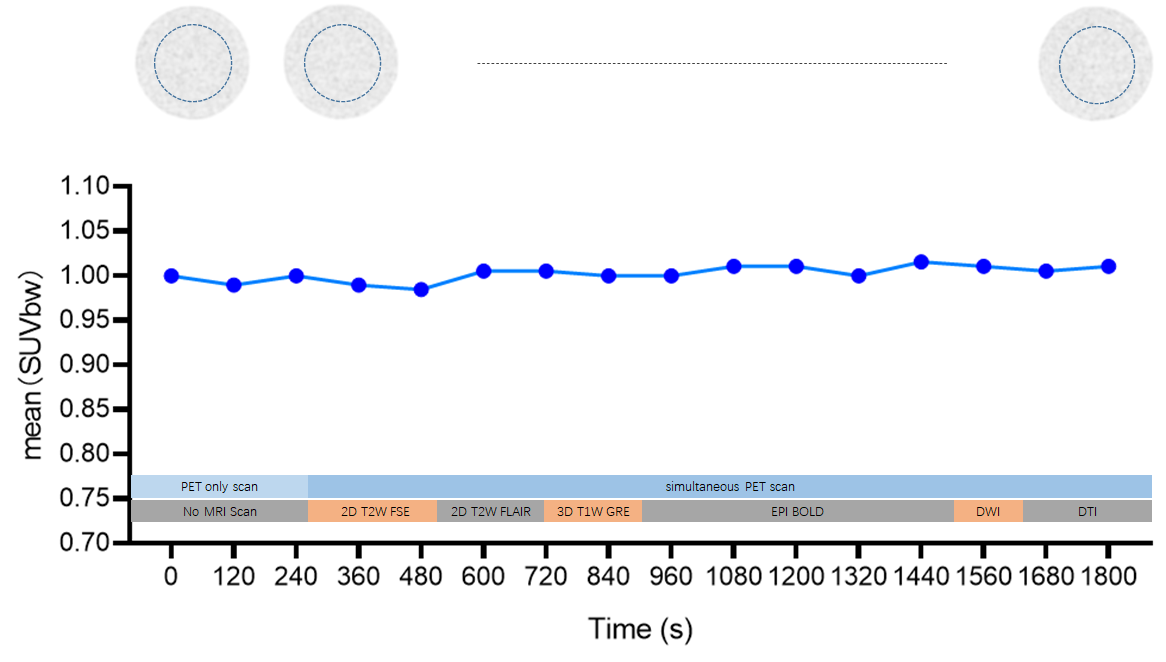
**

FIGURES1: Quantitative evaluation of the SUV stability using the NEMA uniform FDG phantom in the setting of PET only scan in the first 6 minutes and simultaneous PET/MR scan. The mean of SUV was calculated from the ROI (blue dot circle) on the center slice of uniform phantom.

Table S1. MR SNR measurement with and without simultaneous PET acquisition

| MR Sequence | SNR _PETon_ | SNR_PEToff_ | Difference |
| --- | --- | --- | --- |
| 2D T2W FSE | 259 | 258 | 0.46% |
| 2D T2W FLAIR | 327 | 328 | -0.32% |
| 3D T1W GRE | 1041 | 1034 | 0.71% |
| EPI BOLD | 462 | 463 | -0.24% |
| DWI b=0 | 209 | 210 | -0.66% |
| DWI b=1000 | 187 | 186 | 0.60% |
| DTI | 85 | 84 | 1.06% |

SNR, signal noise ratio; FSE, fast spin echo; FLAIR, fluid attenuated inversion recovery; GRE, gradient recalled echo; EPI, echo planar imaging; BOLD, blood oxygen level-dependent; DWI, diffusion weighted imaging; DTI, diffusion tensor imaging.

# MRI Impact on FDG Uptake

To evaluate the effect of reference tissue on the quantitative analysis of SUVr, white matter was selected as the reference as a supplement to the cerebellum as used in the method part. SUVr calculated with reference to white matter reveals higher value than that with cerebellum but demonstrates the same statistical results as found using cerebellum as a reference (Fig. S2).


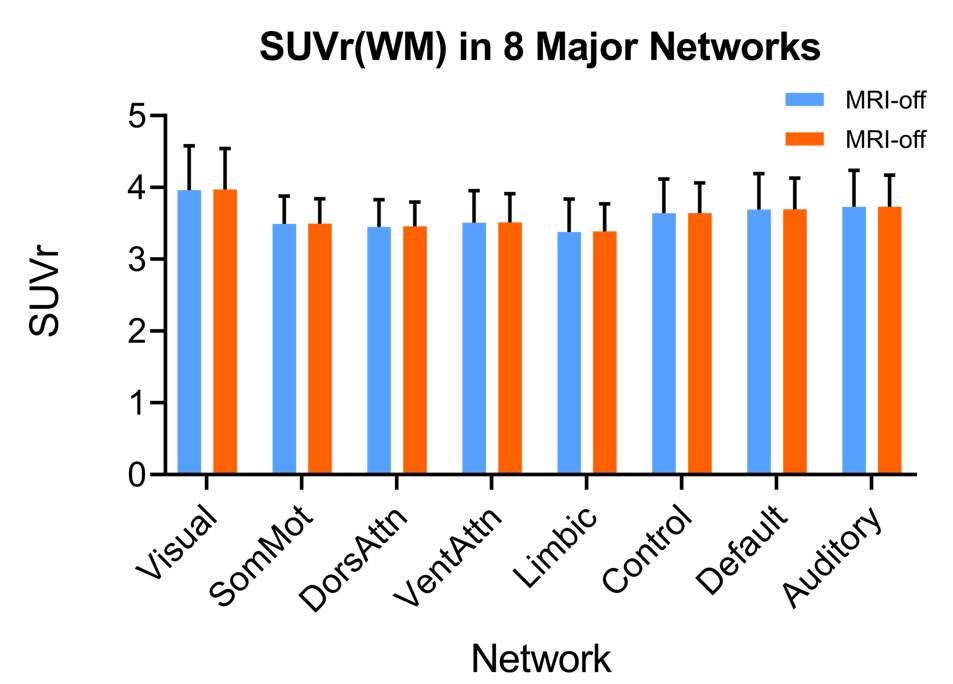


FIGURE S2: Quantitative analysis of static SUVr during the MRI-on and MRI-off mode using white matter as reference tissue.
